# Inhibition of CDC20 potentiates anti-tumor immunity through facilitating GSDME-mediated pyroptosis in prostate cancer

**DOI:** 10.1186/s40164-023-00428-9

**Published:** 2023-08-01

**Authors:** Fei Wu, Minglei Wang, Tao Zhong, Changyan Xiao, Xiaozheng Chen, Yiheng Huang, Meng Wu, Jinming Yu, Dawei Chen

**Affiliations:** 1 Department of Radiation Oncology, Shandong Provincial Key Laboratory of Radiation Oncology, Shandong Cancer Hospital and Institute, Shandong Academy of Medical Sciences, Shandong First Medical University, No.440 Jiyan Road, Jinan, Shandong People’s Republic of China; 2 Research Unit of Radiation Oncology, Chinese Academy of Medical Sciences, Jinan, Shandong China

**Keywords:** CDC20, GSDME, Immunotherapy, Prostate cancer, Pyroptosis

## Abstract

**Background:**

Increasing evidence suggests that immunotherapy, especially immune checkpoint inhibitors (ICIs), has the potential to facilitate long-term survival in various cancer besides prostate cancer. Emerging evidence indicated that pyroptosis, an immunogenic form of cell death, could trigger an anti-tumor immune microenvironment and enhance the effectiveness of immunotherapy. Nevertheless, the mechanism underlying the regulation of pyroptosis signaling in prostate cancer remains unclear.

**Methods:**

The differential expression of human E3 ligases in prostate cancer was integratedly analyzed from five independent public datasets. Moreover, the immunohistochemistry analysis of a tissue microarray derived from prostate cancer patients confirmed the results from the bioinformatic analysis. Furthermore, prostate cancer cell lines were evaluated via the next-generation RNA sequencing to assess transcriptomic profile upon *CDC20* depletion. Next, qRT-PCR, Western blotting, cycloheximide assay, immunoprecipitation, and ubiquitination assay were employed to explore the correlation and interaction between CDC20 and GSDME. Both immune-deficient and immune-competent murine models were utilized to examine the anti-tumor efficacy of CDC20 inhibition with or without the anti-PD1 antibodies, respectively. To analyze the immune microenvironment of the xenografts, the tumor tissues were examined by immunohistochemistry and flow cytometry.

**Results:**

The analysis of multiple prostate cancer cohorts suggested that CDC20 was the most significantly over-expressed E3 ligase. In addition, CDC20 exerted a negative regulatory effect on the pyroptosis pathway by targeting GSDME for ubiquitination-mediated proteolysis in a degron-dependent manner. Knockdown of CDC20 leads to increased GSDME abundance and a transition from apoptosis to pyroptosis in response to death signals. Furthermore, in our syngeneic murine models, we found that depletion of *CDC20* significantly enhances the anti-tumor immunity by promoting the infiltration of CD8^+^ T lymphocytes dependent on the existence of GSDME, as well as reducing myeloid immune cells. More importantly, Apcin, a small molecular inhibitor that targets CDC20, exhibited synergistic effects with anti-PD1-based immunotherapy in murine models of prostate cancer.

**Conclusions:**

Overall, these findings provide new insights into the upstream regulation of GSDME-mediated pyroptosis by CDC20, which specifically interacts with GSDME and facilitates its ubiquitination in a degron-dependent manner. Importantly, our data highlight novel molecular pathways for targeting cellular pyroptosis and enhancing the effectiveness of anti-PD1-based immunotherapy.

**Supplementary Information:**

The online version contains supplementary material available at 10.1186/s40164-023-00428-9.

## Introduction

Increasing evidence suggestingthat immunotherapy, such as immune checkpoint inhibitors (ICIs), chimeric antigen receptor T-cell therapy, and dendritic cell vaccines, has the potential to enable long-term survival in cancer patients [1]. However, the response to immunotherapy varies across tumors and is limited to immunologically “hot” tumors, such as melanoma, non-small cell lung cancer, and bladder cancer [2–4]. Conversely, “cold” tumors, including prostate, pancreatic, and most breast cancers, contain few infiltrating T cells and are less readily recognized by the immune system [5, 6]. Notably, prostate cancer, the most prevalent cancer among men, frequently exhibits resistance to ICIs, underscoringthe necessityfor further studies illustrating the regulation of its immune microenvironment [7, 8].

The tumor microenvironment encompasses diverse environmental and endogenous stressors that could lead to apoptosis in normal cells, such as chronic inflammation, reactive oxygen species, cytokines, and genetic damage [9, 10]. In keeping with that, non-apoptotic cell death, including pyroptosis, autophagy, ferroptosis, and necroptosis, frequently happens in tumors and affects the development of cancer and its response to immunotherapy [11]. Multiple types of non-apoptotic cell death, such as pyroptosis, are defined as immunogenic cell death (ICD) due to the crosstalk between distinct cell death mechanisms and antitumor immunity [12]. Induced by chemotherapy or endogenous stress, the release of damage-associated molecular patterns (DAMPs) and pathogen-associated molecular patterns (PAMPs) during the ICD process promote the development of an inflammatory environment, triggering immune attacks on tumor cells and thereby enhancing antitumor responses [13, 14]. Particularly, pyroptosis plays a pivotal role as a form of ICD for improving the efficacy of cancer immune therapy [15]. Understanding the mechanisms underlying various forms of pyroptosis holds immense importance in the treatment of various cancers [16]. However, the exact role and regulation of pyroptosis signaling in prostate cancer are unclear.

Typically, pyroptosis caused by inflammatory signals occurs in macrophages upon microbial infections and serves a crucial function in the clearance of pathogens. Morphologically, pyroptotic cells are characterized by developing swelling and bubble-like protrusions before eventual cell rupture [17]. As a result, the crack of cells leads to the release of pro-inflammatory cytokines IL-18, IL-1β, and cellular contents into the extracellular space, which could activate an immune response. In the last decade, the pore-forming process’s mechanism during pyroptosis was mediated by the cleavage of gasdermin family members, including GSDMA, GSDMB, GSDMC, GSDMD, and GSDME (DFNA5) [17–21]. For example, the inflammasome-associated caspases (CASP1/4/5/11) could directly cleave GSDMD and promote host defense against infection [17]. More importantly, the GSDME could be cleaved by the apoptosis-dependent caspases (CASP3/8), which play a crucial role in the switching between apoptosis and pyroptosis [18]. In addition, the granzyme B (GZMB) secreted by CD8^+^ T lymphocytes or natural killer cells could cleave the N-terminal fragment of GSDME and form oligomeric death-inducing pores on cell membranes [22]. To this end, the abundance of GSDME determines the alternation between apoptosis and pyroptosis. However, the molecular regulation of GSDME protein in prostate cancer remains elusive.

The dysregulation of cell cycle also characterizes the emergence of cancer. The Anaphase Promoting Complex (APC) and its adaptor subunits, CDC20 and CDH1, have been considered the major driving forces governing cell cycle regulation and tumorigenesis. Previous findings indicated that the oncogenic CDC20 was overexpressed in prostate cancer, partially caused by the frequent mutation of Speckle-type POZ (pox virus and zinc finger protein) protein [23, 24]. As the substrate-recognizing subunit of the APC complex, the CDC20 protein consisted of seven WD40 repeats for binding substrates preserving the destruction box (D-box) motif. However, besides its canonical role in cell cycle progression, the cell cycle-independent functions of CDC20 in prostate cancer remain largely unknown.

As an aberrant expression of E3 ubiquitin ligases can drive cancer progression, CDC20 was found to be the most significantly upregulated E3 ligase among multiple datasets during the screening of 883 human E3-ligases in prostate cancer. Even though known substrates of CDC20 are well documented for their roles in inhibiting prostate cancer development and progression, the exact molecular mechanisms by which CDC20 suppresses anti-tumor immunity in part by regulating pyroptosis have not yet been fully elucidated. To further clarify the physiological role of CDC20 in regulating cell death, we identified the gasdermin protein GSDME as a novel ubiquitin substrate of CDC20 in the prostate cancer setting, which offers further molecular insights into its regulation of anti-tumor immunity.

## Materials and methods

### Animals and cell lines

The experiments in the animal study were conducted using a protocol approved by the Animal Care and Use Committee of Shandong Cancer Hospital, the Shandong First Medical University (Jinan, China). 8 week-old male C57BL/6J mice (specific pathogen-free, SPF) weighing 20 to 25 g were purchased from Beijing Huafukang Bioscience Co. Inc. (Beijing, China). The mice were maintained in an SPF environment at the institutional animal facility (the temperature is 22 ℃, the humidity is 60%, and water and food are available at will, with 12 h of light and 12 h of darkness). Cell lines were obtained from American Type Culture Collection and detected for mycoplasma contamination. HEK293T, PC-3, and VCaP cells were cultured in Dulbecco’s Modified Eagle’s Medium (DMEM) containing 10% fetal bovine serum (FBS), 100 unit/ml penicillin, and 100 mg/ml streptomycin. TRAMP-C2 cells were cultured in a PRMI-1640 medium. Cells were incubated in a humidified incubator with 5% CO2 at 37 °C. Short tandem repeat (STR) profiling has authenticated all cell lines in the past 3 years. In addition, all experiments were performed with mycoplasma-free cells.

### Drugs and therapeutic antibodies

Therapeutic antibodies used in this study were purchased from BioXcell at less than 0.002 endotoxin unit per microgram (2 EU/mg) of endotoxin rat anti-mouse PD-1 (Cat. No. BE0146, Clone: RMP1-14, at 250 µg/dose). The small molecular inhibitor for CDC20, Apcin, was purchased from MedChemExpress (Cat. No. HY-110287). MG132 (Cat. No. A2585), a proteasome inhibitor, was provided by ApexBio Technology, prepared in DMSO solution, and used at a dose of 10 µM. Cycloheximide (CHX), an inhibitor for protein synthesis, was purchased from MedChemExpress (Cat. No. HY-12320). Recombinant Human TNF-alpha protein (rh-TNF-a, 210-TA-020/CF) was purchased from R and D Systems and reconstituted with sterilized PBS (pH 7.4) containing 0.1% BSA.

### Antibodies and plasmids

Constructs of HA-tagged CDC20 and CDH1, lentivirus plasmid containing shRNA targeting human *CDC20* and *CDH1*, and his-tagged ubiquitin were generous gifts from Prof. Jinfang Zhang (Medical Research Institute of Wuhan University). Constructs of flag-tagged wild-type GSDME, shRNA targeting mouse *Gsdme* and *Cdc20*, the N-terminal of CDC20, and WD40 domains of CDC20 were purchased from MiaoLingPlasmid. GSDME deletion degron (RNFL) mutant was generated using the QuikChange II XL Site-Directed Mutagenesis Kit (200521, Stratagene). The antibodies used in immunoblot were listed as follows: mouse monoclonal antibodies against CyclinB1 (sc-245, Santa Cruz, USA), CDC20 (sc-13162, Santa Cruz, USA), CDH1/FZR1 (sc-56312, Santa Cruz, USA), Vinculin (MAB6896, R&D, USA), HA-tag (66006-2-Ig, Proteintech, USA), Flag-tag (66008-4-Ig, Proteintech, USA); rabbit monoclonal antibody against GSDME (ab215191, Abcam); rabbit polyclonal antibody against GSDMD (96458, Cell Signaling Technology, USA), Caspase-3 (9662, Cell Signaling Technology, USA), Cleaved Caspase-3 (9664, 5A1E, Cell Signaling Technology, USA), Cdc20 (10252-1-AP, Proteintech, USA), Actin (AF5003, Beyotime, China), HA-tag (51064-2-AP, Proteintech, USA), Flag-tag (20543-1-AP, Proteintech, USA).

### Prostate tumor treatment experiments

For TRAMP-C2 subcutaneous tumor models,8 weeks old male C57BL/6J mice were allocated randomly into two groups (5 mice per group). A single-cell suspension of TRAMP-C2 cells (5 × 10^5^ cells per animal) infected with indicated shRNAs was suspended in 100 µL serum-free culture and injected into the right upper flank region under aseptic conditions described previously [23]. All tumors were measured by a blinded reader using Vernier calipers every 3 days. The formula used to calculate tumor volumes was as follows: volume(mm^3^) = length (mm) × width(mm)^2^/2. At various experimentally indicated time points after tumor challenge, animals were administrated with Apcin (15 mg/kg/d, i.p. for 4 days per cycle) or anti-PD1 antibodies (10mg/kg, i.p. for twice a week) according to institutional guidelines. 4 weeks later, mice were sacrificed by cervical dislocation, and the tumors were resected and photographed for further analysis.

### Tumor-infiltrating lymphocyte isolation

At 30 days after the TRAMP-C2 tumor challenge, mice were sacrificed under aseptic, and tumors were dissected to investigate the proportion of immune cells by flow cytometry. Briefly, tumor tissue from mice was isolated, minced on ice, and then placed in a culture medium containing DMEM, 2% FBS, 1 mg/ml collagenase 1A (Worthington Biochemical, LS004186), and 0.2 mg/ml DNase I (Sigma-Aldrich, DN25). The cells were incubated with shaking at 37 °C for 30 min, then washed with a 2% FBS cell culture medium (DMEM containing 2% FBS) and filtered with a 70 µm pore size filter. The filtered cell suspension was centrifuged at 800 g for 5 min and washed once with FACS buffer (1 × PBS solution with 2% FBS). The cell pellet was then resuspended and incubated with a diluted solution containing an anti-mouse CD16/32 antibody (Cat. No. 101319, BioLegend, Inc.) to block Fc receptors on immune cells. The cells were centrifuged and washed with FACS buffer, and performed staining of 13 antibodies were at a time (Supplementary Table S5), including Fixable Viability Stain to determine cell viability. The cells were incubated at four °C for 30 min, rinsed with FACS buffer once, and resuspended with FACS buffer. The BD LSR Fortessa flow cytometer was used to detect the fluorescence signal, and the experimental data were further analyzed using FlowJo 10.4 (Tree Star).

### shRNA and plasmid transfection

To generate prostate cancer cells with stable depletion of indicated genes, lentiviral shRNA virus packaging and infection of cell lines were performed according to the protocol described previously [24]. In brief, lentiviral constructs were co-transfected with the pCMVdR8.91 (Delta 8.9) plasmid containing gag, pol, and rev genes, as well as the VSV-G envelope-expressing plasmid into HEK293T cells. Transfection with jetPRIME (Cat. No.101000046, Polyplus Transfection) was

performed based on the manufacturer’s instructions. The supernatant was harvested and filtered with a 0.45 mm syringe filter post-transfection at 48 h and 72 h. The medium containing virus was used for infecting indicated cell lines in the presence of Polybrene (4 mg/ml). Infected cells were selected using 2 µg/ml puromycin dihydrochloride hydrate (A610593-0025, Sangon Biotech) or 300 µg/ml hygromycin B (A100607-0100, Sangon Biotech) for 3 days.

### Reverse transcription and qRT-PCR

RNA was extracted using QIAGEN RN easy mini kit, according to the manufacturer’s instructions (74104, Studio City). For each sample, 1 µg total RNA was reverse transcribed using the iScrip Reverse Transcription Supermix (1708841, Bio‑Rad Laboratories, Richmond, CA, USA). The generated cDNA template was mixed with primers for *GSDME, CDC20, ACTB, and GAPDH*, as well as ChamQ Universal SYBR qPCR Master Mix (Q711, Vazyme), and the real‑time RT‑PCR reaction was conducted with the qTOWER^3^ Real‑Time PCR Detection System (Analytikjena). Data quantitation was performed using the comparative CT (ΔΔCT) method, with GAPDH gene expression as an endogenous reference. The sequences of the indicated primers are listed in Additional file 1: Table S1 

### Immunoblot and immunoprecipitation

Prostate cancer cells were lysed in EBC buffer (50 mM Tris pH 7.5, 120 mM NaCl, 0.5% NP40) supplemented with PMSF (Sigma, Germany) and protease inhibitors (Complete Mini, Roche). The cell lysates were centrifuged at 12,000 g for 10 min at 4 °C. The protein concentrations were measured with the Bradford Protein Assay Kit (Beyotime, China). According to the manufacturer’s instructions, immunoprecipitation assays were performed with the Immunoprecipitation Kit (26149, ThermoFisher). In brief, 1 mg total cell lysates were incubated with the appropriate antibody-conjugated beads (1–2 mg) for 4 h or overnight at 4 ℃. Immunocomplexes were washed 4 times with NETN buffer (20 mM Tris, pH 8.0, 100 mM NaCl, 1 mM EDTA, and 0.5% NP-40). Then immunocomplexes were resolved by SDS-PAGE and immunoblotted with indicated antibodies.

### Lactate dehydrogenase release analysis

Prostate cancer cells with or without depletion of target genes were seeded in 6 well plates and treated as indicated. The supernatant medium was collected and centrifuged at 400×g for 10 min after the administration of drugs. Aliquots of supernatants were transferred into 96-well plates and subjected to the CytoTox 96 non-radioactive cytotoxicity assay kit (Cat. No. G1780, Promega). The percentage of lactate dehydrogenase (LDH) release was calculated as follows: (LDH test – LDH noise) / (LDH maximum − LDH noise) × 100%. The LDH test, LDH background, and LDH maximum are the OD490 of the supernatants from the TNF-α treated, DMSO treated, and lysis buffer (Cat. No. G1780, Promega) treated cells, respectively. Each sample was tested in four replicates to obtain the average levels.

### Analysis of immunohistochemical data

Tissue microarray analysis of CDC20 that contains 102 primary prostate cancers and six normal prostate tissues was purchased from Shanghai Xinchao Company (Shanghai, China). No patients enrolled in the study had received preoperative treatment. The immunohistochemical data of pyroptosis-related proteins were obtained from the Human Protein Atlas database (HPA, https://www.proteinatlas.org/). The immunohistochemical data of patients with prostate cancer and donors as standard control were included and statistically analyzed. To quantify the staining of CDC20 and pyroptosis-related genes, the numerical value for overall intensity (Score A) is based on a 4-point system: 0, 1, 2, and 3 (for none, weak, medium, or intense staining). The numerical value for staining proportion (Score B) is determined by a 5-point system: no stain = 0; ≤25% cells stained = 1; 25–50% cells stained = 2, 50–75% cells stained = 3; all cells stained = 4. Multiply of the scores A and B gives the total score (IHC score). The immunohistochemical staining of CD8A in TRAMPC2 xenografts was analyzed by Image J and its plug-in tools IHC profiler [25].

### Molecular docking

To further explore the protein-protein interaction between CDC20 and GSDME, the X-ray diffraction or predicted structure information was obtained from Protein Data Bank (PDB, https://www.rcsb.org/) and AlphaFoldDB (https://alphafold.ebi.ac.uk/) for molecular docking analysis. ZDOCK (https://zdock.umassmed.edu/), a fast and accurate FFT-based rigid-body docking method utilizing pairwise statistical potentials, was used in this study to investigate the binding ability. Here, the repeated WD40 domains of CDC20 (pdb4ggc, X-ray diffraction, 1.35Å resolution, PDB) and the full-length GSDME (O60443, AlphaFoldDB) were set as the input dataset. The best-scoring decoy was selected and visualized with the PyMOL molecular graphic system (Version 2.6.0a0 Open-Source).

### The dataset used for bioinformatic analysis

We collected datasets from six independent studies in the Gene Expression Omnibus (GEO) database (https://www.ncbi.nlm.nih.gov/geo/), The Cancer Genome Atlas (TCGA) program (https://gdc.cancer.gov/), and Genotype-Tissue Expression (GTEx) project (https://gtexportal.org/home/). All six studies on prostatic disease were obtained by systematically searching literature from the GEO database (https://www.ncbi.nlm.nih.gov/geo/). As a result, four bulk-RNA sequencing studies (GSE70768, GSE28680, GSE6811, GSE2443) were included for integrated analysis. The expression profile of mRNA in cell lines derived from prostate tissues was obtained from the Omics data from the Cancer Cell Line Encyclopedia (CCLE, DepMap Public 22Q2, https://depmap.org/portal/download/). Ethical approval was waived by the institutional ethics committee because data are obtained from public databases, and all the patients are de-identified.

### Analysis of differentially expressed genes

The mRNA expression profile from four bulk-RNA sequencing studies (GSE70768, GSE28680, GSE6811, GSE2443) were included to analyze differentially expressed genes (DEGs). The limma package was used for analysis, linear models, and differential expression, as well as the removal of batch effect of the indicated microarray data. An empirical Bayesian method was conducted to estimate the fold change between the hormone-sensitive prostate cancer (HSPC) group and the castration-resistant prostate cancer (CRPC) group identified by the response to androgen deprivation therapy. The adjusted P-value for multiple testing was calculated using the Benjamini–Hochberg correction. The genes with an absolute log2 fold change more remarkable than two were identified as DEGs between two groups.

### Single sample gene set enrichment analysis

In this study, we evaluated the infiltration of immune cells in prostate cancer from the TCGA dataset using the algorithm of single sample gene set enrichment analysis (ssGSEA) with the R package GSVA (Gene Set Variation Analysis) [26]. Here, the high immune cell infiltration cluster, central immune cell infiltration cluster, and low immune cell infiltration cluster were generated. GSVA was conducted between clusters of different immune cell infiltration states. A heatmap was used to visualize the relationship between the immune infiltration state and the different types of immune cells. The association between gene expression and immune infiltration was visualized in the violin plot. *P* < 0.05 was considered statistically significant.

### Statistics

All data were analyzed using GraphPad Prism software (version 9.0.0). Data were shown as means ± SD (Error bars represent standard deviation). The significance levels for comparison between samples with only two groups were determined by Student’s t-test. One-way ANOVAs followed by Fisher’s least significant difference were applied to test the differences between multiple groups for continuous variables. All images are representative of results from three independent experiments unless otherwise stated. P > 0.05 was considered not significant (ns). The statistical significance computed by the Wilcoxon rank-sum test or t-test is annotated by the number of stars (^*^P < 0.05, ^**^P < 0.01, ^***^P < 0.001).

## Results

### CDC20 was the most significantly altered E3 ligase in prostate cancer and was associated with cell death and immunity

**Fig. 1:**
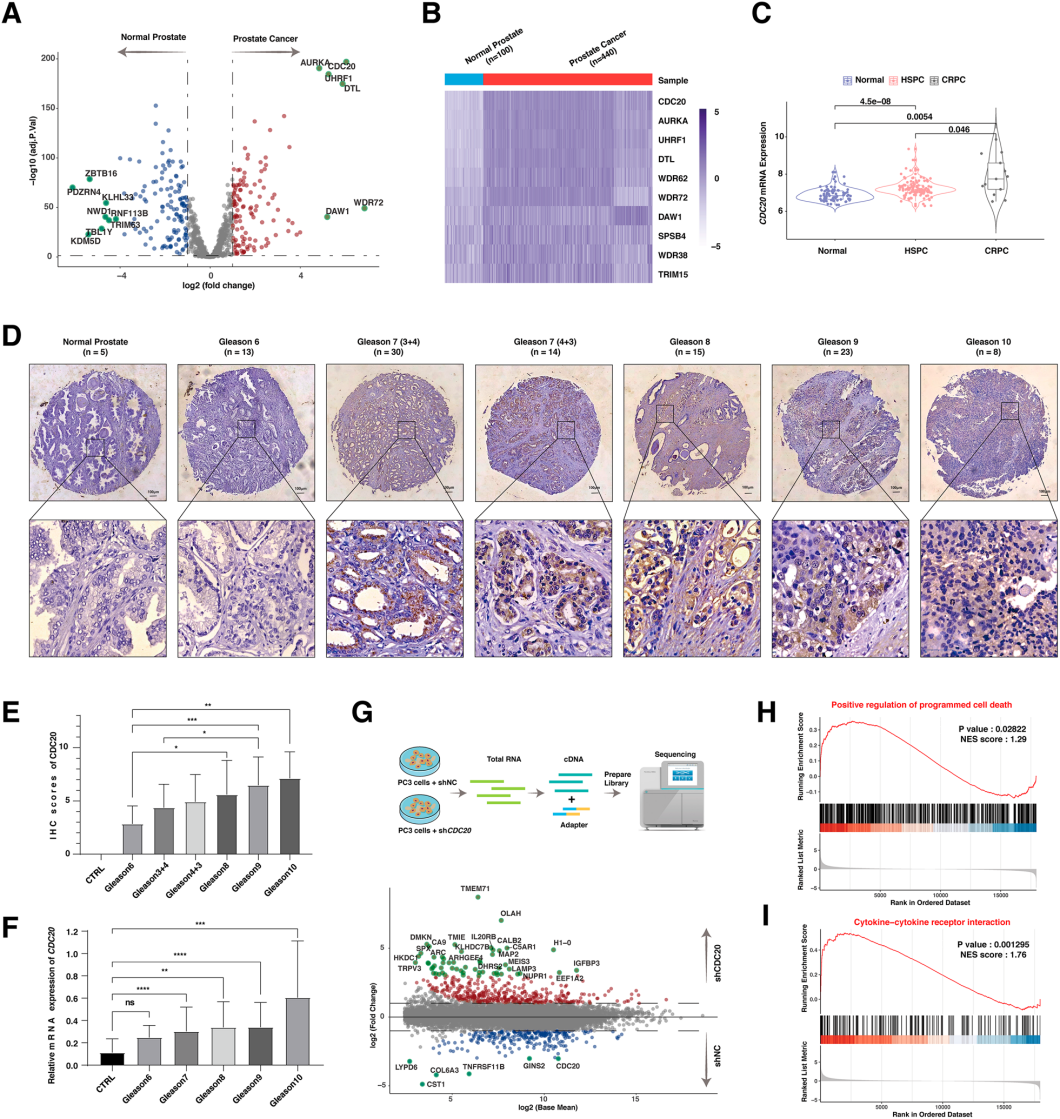
CDC20 was the most significantly altered E3 ligase in prostate cancer and associated with cell death and immunity **A** Volcano plot of the differentially expressed E3 ligases-related genes in TCGA-PRAD datasets. The Red dots show genes that have significant increases in the cancer group. Blue dots show genes that have significant decreases in the cancer group. The heatmap of the top 10 genes in **A**. **C** Heatmap of the top 10 differentially expressed E3 ligase genes in the GSE70768, GSE28680, GSE6811, and GSE2443 datasets. The expression profile of the E3 ligases-related genes was extracted from the indicated datasets and was analyzed among normal prostate, hormone-sensitive prostate cancer (HSPC), and castration-resistant prostate cancer (CRPC). **D **The representative images of immunohistochemistry analysis from 6 normal prostate and 102 prostate cancer tissue with various Gleason scores. The quantification of **D**. **F **The relative mRNA expression of CDC20 in prostate cancer with different Gleason scores in the Oncopression database. **G** A schematic graph indicating the experiment design (up). The volcano plot of the differentially expressed genes in PC3 with or without the depletion of *CDC20*. **H**, **I** GSEA-based GO analysis-enrichment plots of representative gene sets in prostate cancer patients with *CDC20* depletion

Ubiquitination plays many pivotal roles in protein function and degradation. As a result, aberrant expression of E3 ubiquitin ligases can drive cancer progression. To this end, the mRNA expression of 883 human E3-ligases was compared between prostate cancer and normal prostate tissues with dataset from TCGA-PRAD (Fig. 1A, B, Additional file 2: Fig S1, Additional file 1: Table S2. The top 3 most elevated E3 ligases were CDC20, AURKA, and UHRF1 (Fig. 1A, B, Additional file 1: Table S3). Typically, prostate cancer cells were initially dependent on the presence of androgens (HSPC), which became resistant to castration (CRPC) and were biologically invasive after 12 to 24 months. In accordance with the disease progression, the overexpression of CDC20 in HSPC was further elevated in CRPC in four independent studies (The GSE70768, GSE28680, GSE6811, and GSE2443 datasets, Fig. 1C).

CDC20 is also a critical prognosis factor for prostate cancer (Additional file 2: Fig S1E, F). To further confirm the role of CDC20 in prostate cancer, we found that the CDC20 protein abundance was positively correlated with the increased Gleason scores for prostate cancer based on a prostate cancer cohort of 102 cases (Fig. 1D, E, Additional file 1: Table S4). In keeping with that, the association between *CDC20* mRNA expression and the Gleason scores was also significant based on prostate cancer datasets in Oncopression (Fig. 1F)[27]. Next, we sought to understand the role of CDC20 in the transcriptomic profile of prostate cancer by RNA sequencing (PRJNA882665, Fig. 1G). Consequently, the positive regulation of the programmed cell death pathway and cytokine-cytokine receptor interaction pathway was significantly enriched upon the depletion of *CDC20* (Fig. 1H, I).

### The protein stability of GSDME was negatively regulated by CDC20 through the proteasome pathway

**Fig. 2:**
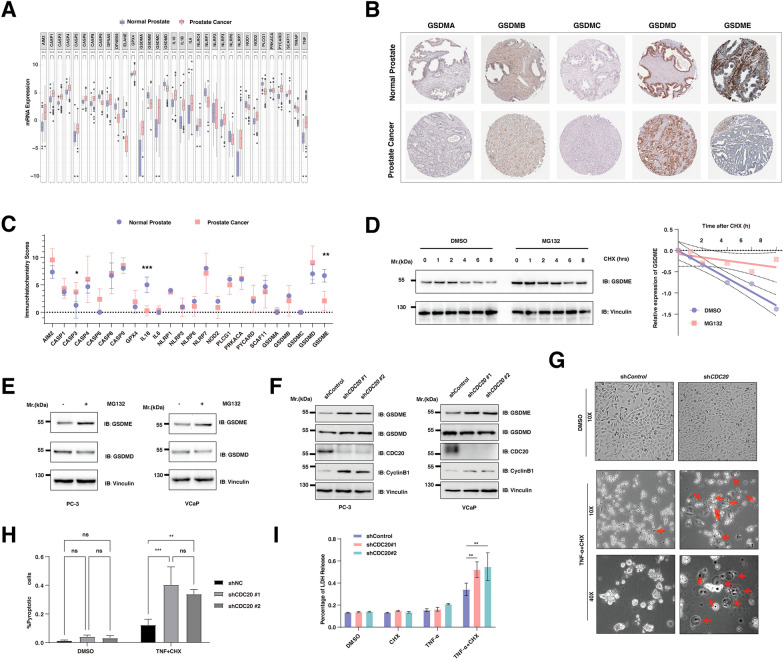
The protein stability of GSDME was negatively regulated by CDC20 through the proteasome pathway. **A** Box plots the differential expression of pyroptosis-related genes between prostate cancer and normal prostate tissues. Pink color labels prostate cancer (n = 499), and the normal blue controls (n = 52). **B**, **C **The immunohistochemical staining (IHC) for protein expression of gasdermin proteins **B**, which were scored and compared statistically **C**. **D** IB analysis of whole cell lysates from PC-3 cells. Cells were harvested at indicated time points after Cycloheximide (CHX) addition with/without MG132 treatment. **E** Immunoblot (IB) analysis of whole cell lysates from PC-3 cells (left) and VCaP cells (right). Cells were harvested 12 h after the addition of DMSO or MG132 treatment. **F** The immunoblot (IB) analysis of whole cell lysates (WCL) derived from PC-3 **B** and VCaP cells **C** infected with the indicated lentiviral shRNA vectors against *CDC20*. **G** PC-3 cells infected with the indicated lentiviral shRNA constructs against *CDC20* for 48 h were selected with 2 µg/ml puromycin for 72 h. The remaining stable cell lines were treated with or without 10 µg/ml cycloheximide (CHX), and 20 ng/ml TNF-α were examined with microscopy the 6 hours post-treatment. **H** The quantification of G. **I** Lactate dehydrogenase (LDH) release in the culture supernatants of PC-3 cells infected with the indicated lentiviral shRNA constructs, treated with DMSO, 10 µg/ml cycloheximide (CHX), or 20 ng/ml TNF-α

In keeping with previous findings, pyroptosis, a critical programmed cell death in cancer, could potentiate immune response. To this end, we explored the expression of pyroptosis-related genes and found that most PRGs were significantly elevated in cancer according to the TCGA-PRAD dataset (Fig. 2A). Specifically, the expression of gasdermins, including *GSDME (DFNA5), GSDMC*, and *GSDMA*, were dominantly elevated (Fig. 2A). Importantly, the protein expression of GSDME, but not other pyroptosis executing gasdermins, was significantly decreased in prostate cancer (Fig. 2B, C). Next, we sought to investigate whether the protein stability of GSDME was affected by the 26S proteasome pathway in prostate cancer. Of note, we found that MG132, a proteasome inhibitor, dramatically prolonged the half-life of endogenous GSDME (Fig. 2D). Furthermore, the abundance of GSDME, but not GSDMD, could be reversed by MG132 (Fig. 2E). As CDC20 was the most significantly upregulated E3 ligase, we firstly depleted *CDC20* in both PC-3 and VCaP cells (Fig. 2F). Our data demonstrated that the protein abundance of GSDME, but not GSDMD, was significantly increased after the depletion of *CDC20* (Fig. 2F). Meanwhile, the mRNA levels of GSDME did not change in the indicated *CDC20* knockdown cells (Additional file 2: Fig. S2). With that, endogenous depletion of *CDC20* significantly switches TNFα-induced apoptosis to pyroptosis in PC-3 cells (Fig. 2G, H). In addition, the deficit of endogenous CDC20 by multiple independent shRNAs constructs increases the release of LDH, indicating pyroptosis's activation (Fig. 2I).

### The protein abundance of GSDME was negatively regulated by APC/CDC20 but not APC/CDH1 in prostate cancer cell lines

**Fig. 3:**
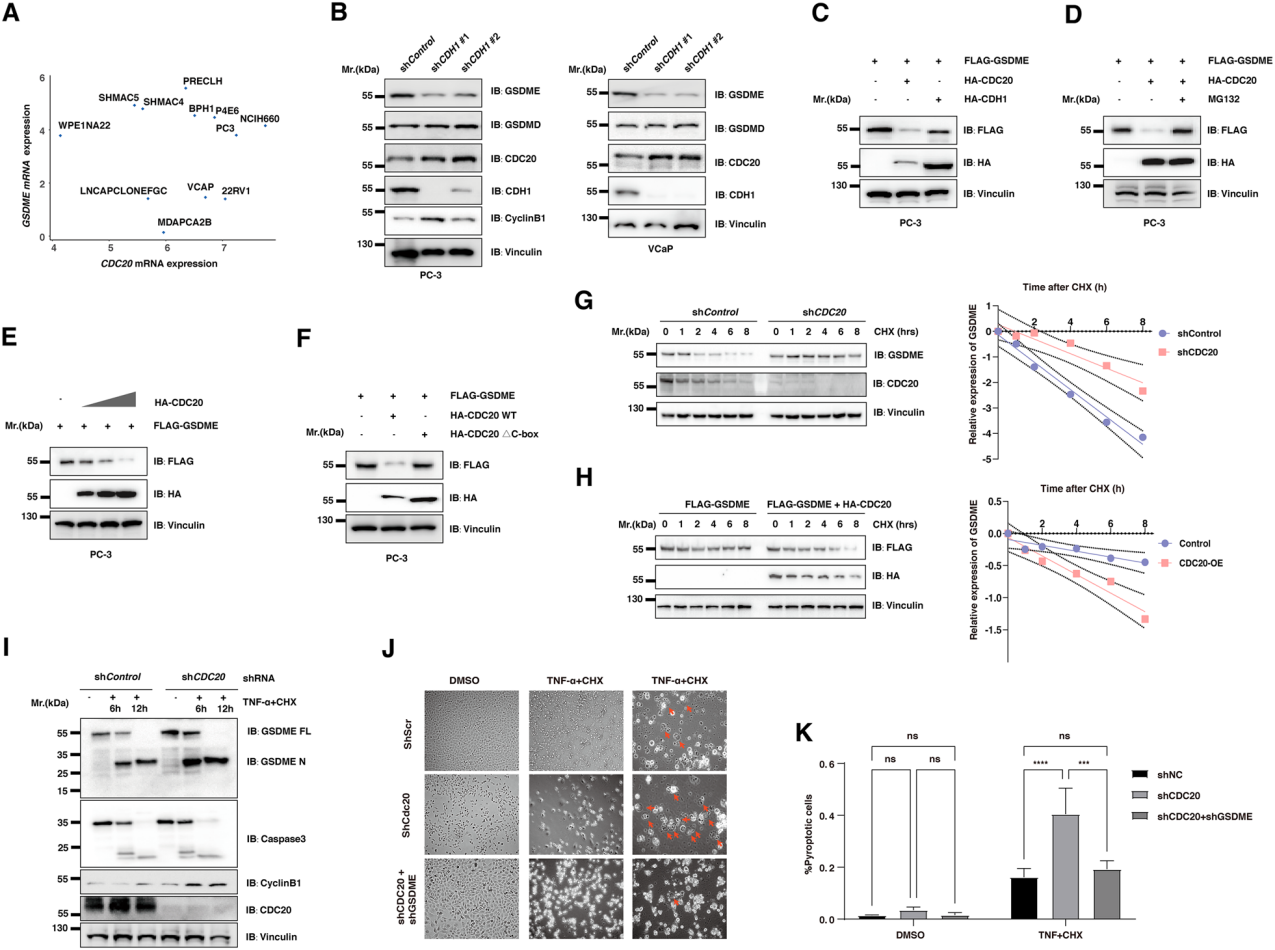
The protein abundance of GSDME was negatively regulated by APC/CDC20 but not APC/Cdh1 in prostate cancer cell lines **A **Scatter plot shows the mRNA expression of *GSDME* and *CDC20* in 12 cell lines derived from the prostate-based CCLE. **B** The IB analysis of WCL derived from PC-3 **D** and VCaP cells **E** infected with the indicated lentiviral shRNA vectors against *CDH1*. **C** The IB analysis of WCL derived from PC-3 cells transfected with the constructs of Flag-GSDME, HA-CDC20, and HA-CDH1. **D** The IB analysis of WCL derived from PC-3 cells transfected with the constructs of Flag-GSDME and HA-CDC20 with or without the addition of MG132. **E** The IB analysis of WCL derived from PC-3 cells transfected with the constructs of Flag-GSDME, HA-CDC20. **F** The IB analysis of WCL derived from PC-3 cells transfected with the constructs of Flag-GSDME, wild-type CDC20 (HA-CDC20 WT), and its degrons deleted mutants (HA-CDC20 ΔC-box). **G** PC-3 cells infected with the indicated lentiviral shRNA constructs against *CDC20* for 48 h were selected with 2 µg/ml puromycin for 72 h. The remaining stable cell lines were treated with 100 µg/ml cycloheximide (CHX) and were harvested at the indicated time points. **H** PC-3 cells were transfected with the indicated constructs. 30 h post-transfection, cells were treated with 100 µg/ml cycloheximide (CHX) and were harvested at the indicated time points. **I** PC-3 cells infected with the indicated lentiviral shRNA constructs against *CDC20* for 48 h were selected with 2 µg/ml puromycin for 72 h. The remaining stable cell lines were treated with or without 10 µg/ml cycloheximide (CHX), and 20 ng/ml TNF-α were harvested at the indicated time points. **J** PC-3 cells infected with the indicated lentiviral shRNA constructs against *CDC20* or *GSDME* for 48 hours were selected with 2 µg/ml puromycin for 72 h. The remaining stable cell lines were treated with or without 10 µg/ml cycloheximide (CHX), and 20 ng/ml TNF-α were examined with microscopy the 6 h post-treatment. **K** The quantification of G

To further examine this hypothesis in prostate cancer cell models, we found that PC3, NCIH660, VCAP, and 22RV1 expressed CDC20 and GSDME at high levels with dataset from CCLE (Fig. 3A). Furthermore, depletion of *CDH1*, a closely related family member of CDC20, failed to increase the abundance of GSDME (Fig. 3B). Significantly, the ectopic overexpression of CDC20, but not CDH1, promoted the destruction of GSDME in a dose-dependent manner, which could be reversed by MG132 (Fig. 3C–E). Furthermore, deletion of the catalytic subunit of CDC20, named the CDC20 △C-box, failed to affect the stability of GSDME (Fig. 3F). In support of this notion, CDC20 depletion led to a significant increase in the stability of GSDME in the presence of cycloheximide (CHX) (Fig. 3G). Meanwhile, ectopic overexpression of CDC20 decreased the stability of GSDME with the administration of CHX (Fig. 3H). Previous studies have proved that GSDME could be cleaved by the activated caspase-3 followed by GSDME-dependent pyroptosis. In further support of a physiological role for CDC20 in governing pyroptosis, the TNF-α treatment assay in PC3 cells showed that depletion of *CDC20* could significantly increase the abundance of the full-length GSDME (GSDME FL), as well as the cleaved N-term of GSDME (GSDME N), upon the stimulation of TNF-α and CHX (Fig. 3I). Accordingly, endogenous depletion of *CDC20* significantly switches TNFα-induced apoptosis to pyroptosis in PC-3 cells, which could be reversed by additional depletion of GSDME (Fig. 3J, K).

### The conservative RNFL sequence in GSDME specifically interacts with the repeated WD40 domain of CDC20

**Fig. 4:**
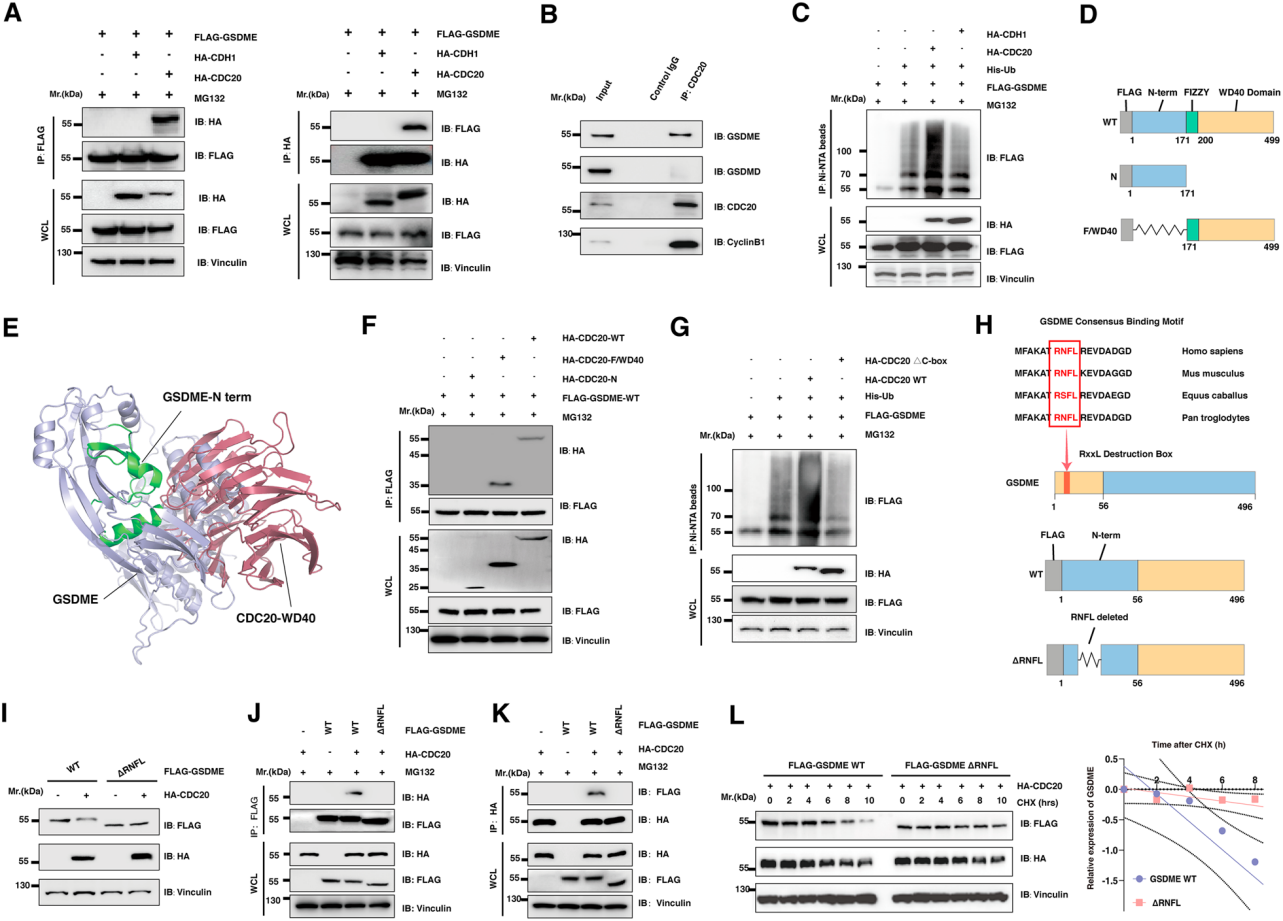
The conservative RNFL sequence in GSDME specifically interacts with the repeated WD40 domain of CDC20 **A** Immunoblot (IB) analysis of whole cell lysates (WCL) and immunoprecipitation (IP) derived from 293T cells transfected with indicated constructs. Then cells were administered 10 µM MG132 for 12 h before harvesting. **B** Immunoblot analysis of whole cell lysates (Input) and IP derived from wild-type PC-3 cells. Then cells were administered 10 µM MG132 for 12 h before harvesting. ** C** IB of WCL and His pull-down of PC-3 cells transfected with the indicated constructs. Then cells were treated with 10 µM MG132 for 12 h and lysates with denaturing buffer. **D** Schematics of indicated CDC20 constructs with or without the deletion of WD40 and Fizzy domains or N-term. ** E** Molecular docking studies of GSDME full length against WD40 domain of CDC20. Protocol validation of molecular docking experiment using ZDock and PyMOL. Representative images of the predictive binding model were visualized. **F** IB of WCL and IP derived from 293T cells transfected with indicated constructs. **G** IB of WCL and His pull-down of PC-3 cells transfected with the indicated constructs. Then cells were treated with 10 µM MG132 for 12 h and lysates with denaturing buffer. **H** Sequence comparison of putative CDC20 binding motif in GSDME with known CDC20 substrates. CDC20 binding consensus sequences are shown in red. Schematics of indicated GSDME constructs with or without the RNFL consensus binding motif deletion. **I** The IB analysis of WCL derived from PC-3 cells transfected with the constructs of Flag-GSDME and HA-CDC20. **J**–**K** IB of WCL and IP derived from 293T cells transfected with indicated constructs. Then cells were treated with 10 µM MG132 for 12 h before harvesting. **L** PC-3 cells were transfected with the constructs as indicated. 30 h post-transfection, cells were treated with 100 µg/ml cycloheximide (CHX) and were harvested at the indicated time points

In keeping with these findings, GSDME specifically interacts with CDC20, but not CDH1, both in vitro and in vivo (Fig. 4A, B). Consistently, the wild-type CDC20, but not CDH1, promoted the in vivo polyubiquitylation of recombinant GSDME (Fig. 4C). To gain further mechanistic insights into the physiological role of CDC20 in modulating GSDME abundance, we generated multiple CDC20 constructs expressing wild-type CDC20, the truncated N-term of CDC20, and the truncated WD40 domain CDC20 (Fig. 4D). Firstly, molecular docking studies of GSDME full length against the WD40 domain of CDC20 further support the protein binding between GSDME and CDC20 (Fig. 4E). Moreover, CDC20 mutant deleting the WD40 repeat domain failed to interact with GSDME (Fig. 4F). In addition, the wild-type CDC20, but not CDC20 mutant deleting the WD40 repeat domain of CDC20 with C-box deletion, promoted the in vivo polyubiquitylation of GSDME (Fig. 4G). Together, these results demonstrate that CDC20 specifically interacts with GSDME.

Several identified CDC20 substrates, including CylinB1, share a CDC20-binding consensus motif RXXL (Fig. 4H). Here, in the N-term of GSDME, the functional pore-forming domain of GSDME, we found a conservative ^7^RNFL^10^ consensus motif, which was hypothesized to be the destruction box that interacts with CDC20. To further investigate the role of ^7^RNFL^10^ motif in GSDME, we generated GSDME constructs expressing wild-type GSDME, the truncated GSDME with ^7^RNFL^10^ deletion (Fig. 4H). Notably, the GSDME mutant with RNFL deletion became resistant to CDC20-mediated destruction (Fig. 4I). We consistently identified that deleting this putative motif (GSDME-△RNFL) impaired GSDME interaction with CDC20 in cells (Fig. 4J, K). In addition, ectopic overexpression of GSDME-△RNFL had increased protein stability compared with the wild-type GSDME (Fig. 4L). Taken together, these results demonstrate that the ^7^RNFL^10^ motif is required for GSDME to interact with CDC20 and subsequently triggers poly-ubiquitination and degradation of GSDME.

### CDC20 remodels an immune-suppressive microenvironment through GSDME

**Fig. 5:**
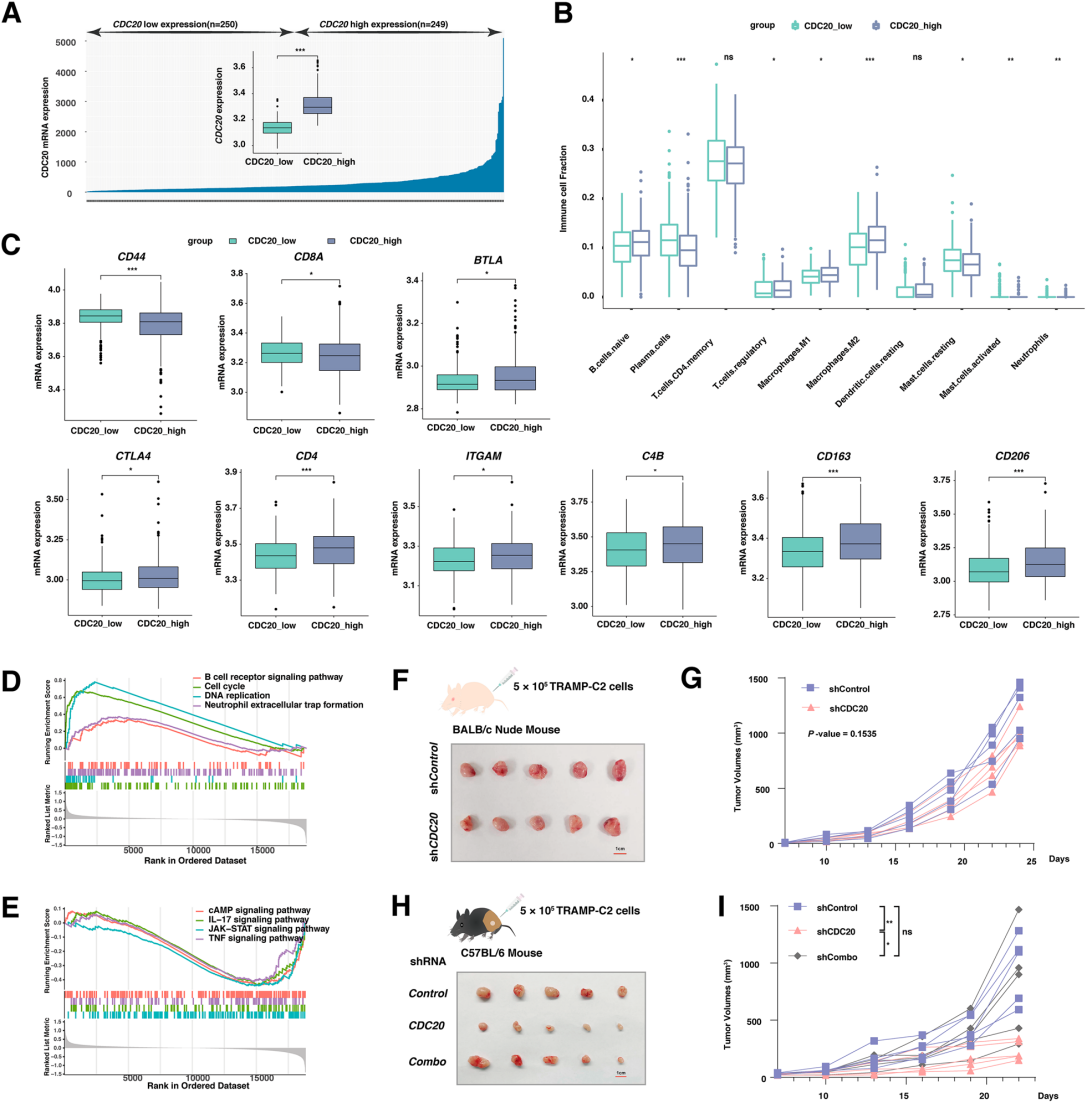
CDC20 remodels an immune-suppressive microenvironment through GSDME **A** The mRNA expression of CDC20 was compared between the CDC20 low and high groups in the prostate cancer patients from the TCGA-PRAD datasets. **B** Box plot analysis exhibiting distinct infiltration of immune cells subpopulation in prostate cancer at different CDC20 expression levels (CDC20_low and CDC20_high). **C** Box plots show the statistical comparison of the mRNA expression of marker genes in various immune cells at different CDC20 expression levels (CDC20_low and CDC20_high). **D–E** GSEA-based GO analysis-enrichment plots of representative gene sets, which activated **D** or suppressed **E** in prostate cancer patients with *CDC20*-high expression. The colored line means enrichment profile. **F** The schematic graph and the picture of xenografts after the sacrifice of BALB/c mice bearing TRAMP-C2 tumors. **G** The volume of xenografts in BALB/c mice bearing TRAMP-C2 tumors was measured every 3 days from the 7th day after implantation (5 mice per group). **H** The schematic graph and the picture of xenografts after the sacrifice of C57BL/6 mice bearing TRAMP-C2 tumors infected with the indicated lentiviral shRNA constructs. **I** The volume of xenografts in C57BL/6 mice bearing TRAMP-C2 tumors infected with the indicated lentiviral shRNA constructs was measured every 3 days from the  7 day after implantation (5 mice per group)

Next, we sought to understand the role of the CDC20-GSDME interaction in the immune microenvironment in prostate cancer. Firstly, the cases from the TCGA-PRAD datasets were divided into two groups based on the mRNA expression of *CDC20* (Fig. 5A). Deconvolution analysis of bulk-transcriptomic data indicated that the M2-macrophages and regulatory T cells were significantly increased in the *CDC20*-high group. In contrast, plasma cells and mast cells were decreased (Fig. 5B). In keeping with that, the markers for anti-tumor immunity, including *CD44* and *CD8A* were significantly reduced in the *CDC20*-high group (Fig. 5C). Consistently, the features for suppression of anti-tumor immunity were dominantly elevated in the *CDC20*-high group, such as *BTLA, CTLA4, CD4, ITGAM, C4B, CD163*, and *CD206* (Fig. 5C). GSEA analysis showed that the pathways in cells cycles and DNA replication were primarily increased in *CDC20-*high group (Fig. 5D, Additional file 1: Table S5). Significantly, multiple signaling pathways of immune activation were enriched in the *CDC20-*low group, including IL-17, JAK-STAT, and TNF signaling pathways (Fig. 5E). To gain further insight into the role of CDC20 in anti-tumor immunity, and we generated multiple TRAMP-C2 cell lines with various independent shRNAs constructs targeting CDC20 or GSDME. For the xenografts in BALB/c mice bearing TRAMP-C2 tumors, the tumor growth curve had no significant difference between the control group and the CDC20-depleted groups (Fig. 5F–G). However, in the immune-competent C57BL/6 mice, the TRMP-C2 xenografts with depletion of CDC20 significantly decreased compared with the control group, which could be reversed by the additional depletion of GSDME (Fig. 5H–I).

### CDC20 promotes the response to immune checkpoint inhibitors and the activation of anti-tumor immunity through GSDME

**Fig. 6:**
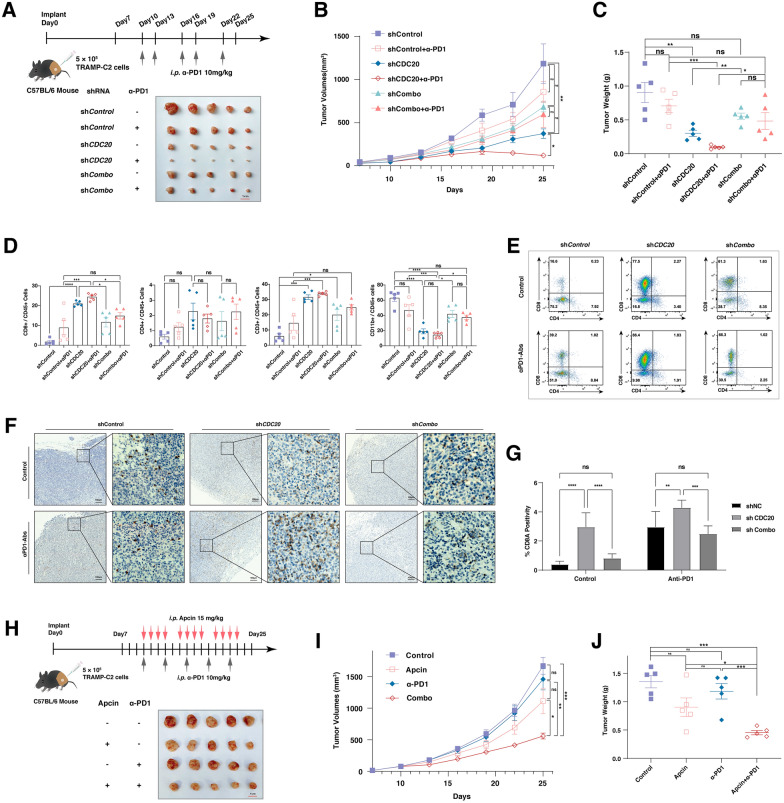
CDC20 promotes the response to immune checkpoint inhibitors and the activation of anti-tumor immunity through GSDME **A** The schematic graph of experimental design and the picture of xenografts after the sacrifice of mice. Mice bearing TRAMP-C2 tumors infected with the indicated lentiviral shRNA constructs were implanted 7 days earlier and treated with or without anti-PD1 monoclonal antibodies (5 mice per group). **B** The volume of xenografts in C57BL/6 mice bearing TRAMP-C2 tumors infected with the indicated lentiviral shRNA constructs with or without administering anti-PD1 monoclonal antibodies. **C** The tumor weight of xenografts after the sacrifice of indicated mice in A. **D** Flow cytometric analysis of the single cell suspension derived from TRAMP-C2 tumors in A. **E** Representative flow cytometric analysis figures indicate the difference between CD8^+^ and CD4^+^ T lymphocytes in TRAMP-C2 tumors implanted in C57BL/6 mice. **F** The immunohistochemistry staining for CD8 on TRAMP-C2 tumors implanted in C57BL/6 mice infected with the indicated lentiviral shRNA constructs with or without anti-PD1 monoclonal antibodies. **G** The quantification of F. **H **The schematic graph of experimental design and the picture of xenografts after the sacrifice of mice. Mice bearing TRAMP-C2 tumors implanted 7 days earlier were treated with or without the Cdc20 inhibitor, Apcin, or the anti-PD1 monoclonal antibodies (5 mice per group). **I** The volume of xenografts in C57BL/6 mice bearing TRAMP-C2 tumors with or without administering anti-PD1 monoclonal antibodies or Apcin **J** Xenografts’ tumor weight after the sacrificed indicated mice in **H**

**Fig. 7:**
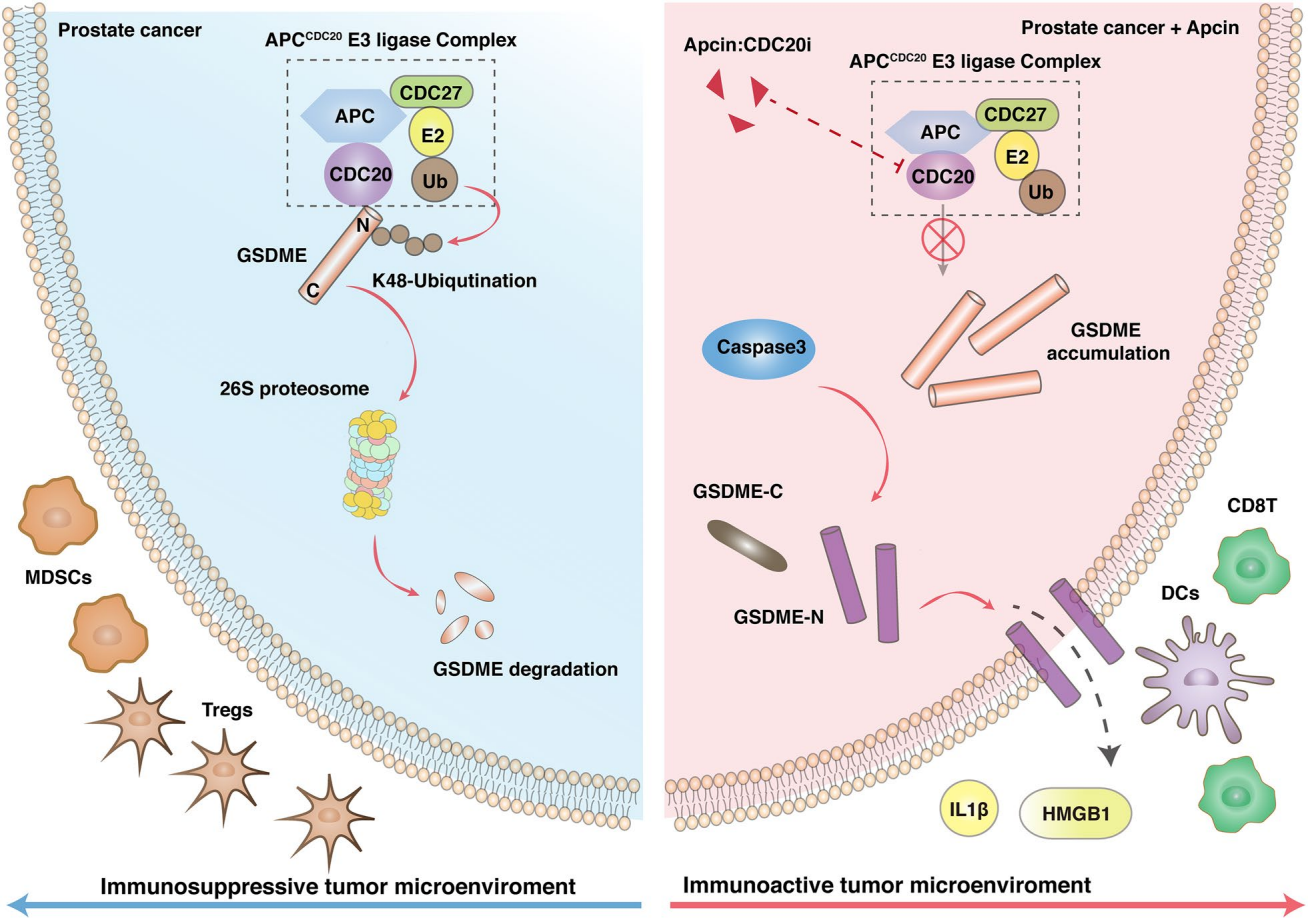
Flow diagram** A** schematic graph indicates the interaction between CDC20 and GSDME in the pathogenesis of prostate cancer with or without the administration of Apcin

To explore the physiological roles of CDC20-mediated GSDME degradation in vivo, we found that decreased growth of TRAMP-C2 tumors implanted in the immune-competent C57BL/6 mice caused by the depletion of *CDC20* could be significantly reversed by the additional lack of *GSDME* (Fig. 6A–C). In keeping with that notion, the depletion of CDC20 dramatically enhanced the role of anti-PD1 antibodies in the TRAMP-C2 context, which became less significant with the additional knockdown of GSDME (Fig. 6A–C). In our murine transplantable preclinical models, the CD3^+^ lymphocytes, but not the myeloid cells, significantly increased in the xenografts with CDC20 depletion, despite the administration of anti-PD1 antibodies (Fig. 6D, Additional file 2: Fig. S3, Table S6). Specifically, the CD8 + T lymphocytes dominantly increased in CDC20 depleted groups, while the CD4^+^ T lymphocytes failed to have significant differences (Fig. 6E). Consistently, the increase of CD8^+^ T lymphocytes upon CDC20 depletion could be reversed by the additional deletion of GSDME (Fig. 6E). In keeping with that, examination of the CD8A staining through TRAMP-C2 tumors with or without the depletion of *CDC20* or *GSDME* following ICIs therapy revealed prominent CD8^+^ T cells infiltration upon *CDC20* depletion (Fig. 6F, G). To gain further evidence of the previous findings and explore the translational potential of CDC20 targeting therapy, Apcin, a molecular inhibitor of CDC20, was used for competitive binding and suppression of functional CDC20. Here, the combination of Apcin with α-PD-1 showed prominent therapeutic superiority to α-PD-1 alone (P < 0.01) and to Apcin alone (P < 0.01) (Fig. 6H–J). Above all, depletion of CDC20 enhances the anti-tumor immunity and synergistic effects with α-PD-1 mediated by GSDME (Fig. 7). To extend these finding, using TIDE (Tumor Immune Dysfunction and Exclusion), we analyzed the relationship between survival fraction and cytotoxic T lymphocyte (CTL) levels in two patient groups: CDC20-low and CDC20-high (Additional file 2: Fig. S4, Additional file 1: Table S8). As a result, the survival benefits of increased CTL levels were more likely to be occurred in the CDC20-high cases, but not the CDC20-low patients in melanoma, lung cancer, breast cancer, as well as head and neck cancer (Additional file : 2Fig. S4).

## Discussion

A significant impediment to the overall effectiveness of immune checkpoint inhibitors is the illusive mechanism of immune suppression in “cold” tumors associated with poor prognosis. In this study, we investigated the molecular characteristics of prostate cancer, a well-known “cold” tumor in immune therapy, and explored the physiological links between pyroptosis-related genes and the anti-tumor immunity in prostate cancer. Our data indicated that CDC20, an E3 ligase highly overexpressed in both prostate cancer and CRPC, negatively regulated the pyroptosis pathway by targeting GSDME for ubiquitination-mediated proteolysis in a degron-dependent manner. Moreover, we demonstrate that depletion of CDC20 by shRNA constructs or small molecular inhibitors significantly enhances the anti-tumor immunity dependent on the existence of GSDME in prostate cancer and exhibits synergistic effects when combined with α-PD-1 therapy. These results add a new layer to understanding the upstream controlling of GSDME and unveil novel molecular pathways for therapies targeting the pyroptosis pathway.

CDC20 and CDH1 are the essential adaptor proteins of the anaphase-promoting complex, playing a critical role in regulating mitosis and oncogenesis [28]. Preclinical exploration have shown that specific CDC20 inhibitors, including proTAME and Apcin, are effective against multiple types of malignant tumors, resulting in mitotic arrest and apoptosis and synergizing with clinically-relevant drugs [29–31]. Here, our data found that CDC20, but not CDH1 could specifically and negatively target GSDME for ubiquitination and degradation. Our data showed that CDC20 expression is elevated in prostate cancer compared with normal prostates and is further upregulated CRPC following disease progression. In addition, the protein abundance of CDC20 was negatively regulated by Cullin3^SPOP^ complex, with SPOP being one of the most frequently mutated genes in prostate cancer [24, 32]. To this end, the increase of functional CDC20 protein in prostate cancer patients could be higher than the fold changes at transcriptional levels in practical clinical settings. As a result, we hypothesized that the molecular targeting CDC20 could exhibit potent specificity and efficiency. In addition, it has been reported that the activation of CDK1/SPOP signaling by the ATR inhibition enhanced the cytotoxicity of ICIs in prostate cancer [32]. Given that SPOP negatively regulates CDC20, it is plausible that the inhibition of CDC20 and increase of GSDME could enhance ICIs efficiency based on findings in this study. Currently, several clinical trials utilizing ICIs treatment for prostate cancer have not achieved significant survival benefits [33]. Moreover, these trials lack sequencing information from patient samples, thereby limiting the exploration of the correlation between CDC20 expression levels and immunotherapy efficacy.Nevertheless, more preclinical models, such as transgenic murine models of prostate cancer, are needed to confirm further the effects of CDC20 depletion and the synergistic effects with various ICIs.

To date, the cancer treatment strategy has switched from eliminating the entire tumors barely through radiation or medicine to achieving long-term survival by suppressing tumors through activation of the inherent immune system [34]. Unlike non-immunogenic apoptosis, which lacks the ability to trigger an inflammatory response, pyroptosis is an immunogenic cell death that can instigate local inflammation. This process facilitates the infiltration of immune cells, providing an promising avenue for mitigating the immunosuppression of tumor microenvironments [11, 22]. However, canonical pyroptosis due to the cleavage of GSDMD usually occurs in macrophages upon infection of exogenous pathogens, which is essential for pathogen clearance [11]. Recently, the cleavage of GSDME by caspase three was also reported to be one of the most critical signalings in forming pyroptosis in cancer [18]. Notably, the protein abundance of GSDME governs the switch of cell death types induced by chemotherapeutic drugs. For instance, cancer cells with highly expressed GSDME protein were dominantly undergoing pyroptosis upon the death signals, while GSDME-low cancer cells underwent apoptosis. Thus, it is vital to illustrate the upstream regulation of GSDME to further utilize the immunogenic effects of pyroptosis to boost the anti-tumor effects of immune therapy. Our study reported that the GSDME could be negatively regulated by an oncogenic protein, CDC20, in prostate cancer. Furthermore, our data collectively demonstrated that the role of CDC20 depletion in enhancing the infiltration of anti-tumor immune cells, particularly CD8^+^ T lymphocytes, and this effect is dependent on the presence of GSDME. Since CDC20 depletion led to the increase of pyroptosis cells formation and LDH release in vitro, we postulated that the enhanced anti-tumor immunity could be attributed to the release of cellular contents, including cytokines IL-1β, IL-18, HMGB1, and other contents, into the extracellular space, subsequently triggering local inflammation. Although several small molecular inhibitors targeting CDC20 have been documented in the literature, their roles in combination with ICIs have not been thoroughly investigated [31]. To this end, future studies are warranted to systematically explore the potential of this pyroptosis-dependent therapy by targeting CDC20.

Importantly, IL-18, one of the cytokines released upon pyroptosis, is a pro-inflammatory cytokine that has been implicated in prostate cancer [35]. Previous studies have suggested that IL-18 expression in prostate cancers was associated with a favorable outcome [36]. Interestingly, IL-18 was also upregulated at the mRNA level but downregulated at the protein level in our dataset. This discrepancy suggests that post-transcriptional regulation may play a role in modulating IL-18 expression in prostate cancer. The precise mechanisms underlying this downregulation remain unclear, but potential explanations include altered protein stability or the activity of microRNAs that target IL-18 mRNA for degradation. Regardless of this apparent discordance between mRNA and protein levels, it is important to note that the observed downregulation of IL-18 at the protein level could still have functional consequences.

## Conclusions

Taken together, our findings unveil a negatively regulating mechanism toward GSDME by CDC20 in controlling pyroptosis signaling, indicating that the molecular targeting of CDC20 can modulate the anti-tumor immunity and the synergistic effects with α-PD-1 therapy in prostate cancer in the presence of GSDME. These results add a new layer to understanding the upstream controlling of GSDME-mediated pyroptosis and providing novel molecular pathways that can be targeted for cancer immunotherapy.

## Supplementary information

**Additional file 1: Table S1.** List of primers for qRT-PCR. **Table S2.** The list of human E3 ligases. **Table S3.** The differential expressing E3 ligase genes between the prostate cancer and normal prostate. **Table S4.** The analysis of clinical characteristics and the expression of CDC20 by immunohistochemistry. **Table S5.** The differential expressing genes between the CDC20-low and CDC20-high group. **Table S6.** List of FACS antibodies. **Table S7.** Detailed information of datasets used in this study. **Table S8.** The high confidence results (Core) and CDC20 Expression from TIDE analysis.

**Additional file 2: Figure S1.**Exploratory analysis of TCGA data focused on Cdc20 in prostate cancer. **Fig. S2.** Relative mRNA expression of *CDC20* (A) and *GSDME* (B) upon depletion of *CDC20. ***Fig. S3.** Fluorescence-activated cell sorting (FACS) gating strategy and analysis of the single cell suspension derived from TRAMP-C2 tumors. **Fig. S4.** Relationship between survival fraction and CTL levels in CDC20-low and CDC20-high patients using TIDE.

## Data Availability

The RNA-seq data generated by this article are available in the NCBI Sequence Read Archive repository with the following SRA accession number: PRJNA882665 (https://dataview.ncbi.nlm.nih.gov/object/PRJNA882665?reviewer=pajtovqansv9722kngimlpdhn3). The Accession number for the bulk-RNAseq are GSE70768, GSE28680, GSE6811, and GSE2443. The pan-cancer datasets used in this study can be obtained and downloaded from XENA (https://xenabrowser.net/). The datasets and scripts supporting the results will be available from the corresponding author upon request.

## Abbreviations

ICIs: Immune checkpoint inhibitors
ICD: Immunogenic cell death
APC: Anaphase-promoting complex
D-box: Destruction box
IHC: Immunohistochemical
DMEM: Dulbecco's Modified Eagle's Medium
FBS: Fetal bovine serum
STR: Short tandem repeat
CHX: Cycloheximide
FACS: Fluorescence activating cell sorter
PDB: Protein Data Bank
GEO: Gene expression Omnibus
TCGA: The cancer genome Atlas
GTEx: Genotype-tissue expression
CCLE: Cancer cell line Encyclopedia
DEGs: Differentially expressed genes
HSPC: Hormone-sensitive prostate cancer
CRPC: Castration-resistant prostate cancer
ssGSEA: Single sample gene set enrichment analysis
GSVA: Gene set variation analysis
SD: Standard deviation
LDH: Lactate dehydrogenase
