# Supplementary material for: Inhibition of CDC20 potentiates anti-tumor immunity through facilitating GSDME-mediated pyroptosis in prostate cancer
Source: Exp Hematol Oncol. 2023 Aug 1;12:67. doi: 10.1186/s40164-023-00428-9 (PMC10391908; doi:10.1186/s40164-023-00428-9)
Supplement: Supplementary file 2 — Additional file 2: Figure S1.Exploratory analysis of TCGA data focused on Cdc20 in prostate cancer. Fig. S2. Relative mRNA expression of CDC20 (A) and GSDME (B) upon depletion of CDC20. Fig. S3. Fluorescence-activated cell sorting (FACS) gating strategy and analysis of the single cell suspension derived from TRAMP-C2 tumors. Fig. S4. Relationship between survival fraction and CTL levels in CDC20-low and CDC20-high patients using TIDE. [file 40164_2023_428_MOESM2_ESM.doc]

**Additional file Figures for**

**Inhibition of CDC20 potentiates anti-tumor immunity through facilitating GSDME-mediated pyroptosis in prostate cancer**

Fei Wu^1^, Minglei Wang^1^, Tao Zhong^1^, Changyan Xiao^1^, Xiaozheng Chen^1^, Yiheng Huang^1^, Meng Wu^1^, Jinming Yu^1,2*^, Dawei Chen^1*^

*^1^**Department of Radiation Oncology and Shandong Provincial Key Laboratory of Radiation Oncology, Shandong Cancer Hospital and Institute, Shandong First Medical University and Shandong Academy of Medical Sciences, Jinan, Shandong, China*

*^2^Research Unit of Radiation Oncology, Chinese Academy of Medical Sciences, Jinan, Shandong, China*

**^*^**Correspondence should be addressed to:

Dawei Chen (dave0505@yeah.net)

Jinming Yu (sdyujinming@126.com)

Corresponding authors’ address: No.440 Jiyan Road, Jinan, Shandong Province, P.R.China,

Tel: (+86)053187984777, Fax: (+86)053187984079, email: sdyujinming@126.com.

**Running title:** CDC20 inhibition facilitates anti-tumor immunity

**Keywords:** CDC20; GSDME; immunotherapy; prostate cancer; pyroptosis


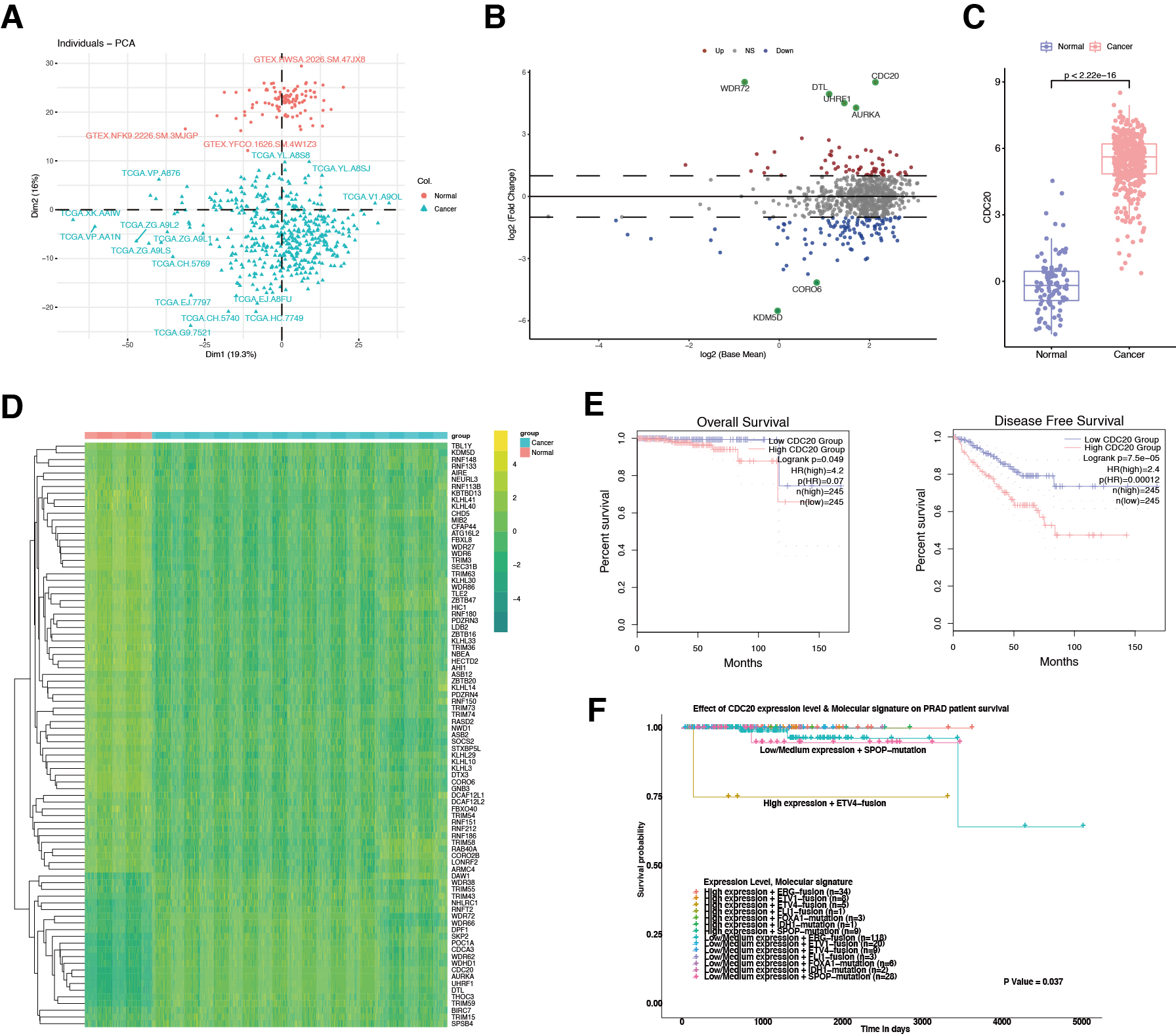


**Additional file 2: Fig.1 Exploratory analysis of TCGA data focused on Cdc20 in prostate cancer**

1. Principal component analysis of the RNA sequencing dataset derived from prostate cancer patients in The Cancer Genome Atlas Prostate Carcinoma (TCGA-PRAD) and normal prostate tissues in Genotype-Tissue Expression (GTEX).
2. Ratio intensity (MA) plot of the differentially expressed E3 ligases related genes in TCGA-PRAD datasets. The Red dots show genes that have significant increases in the cancer group. Blue dots show genes that have significant decreases in the cancer group.
3. Box plots show the statistical comparison of *CDC20* mRNA expression among tumor and normal groups TCGA-PRAD datasets.
4. Heatmap shows the top100 differential gene expression among tumor and normal groups TCGA-PRAD datasets.
5. Comparison of the overall survival (left) and disease-free survival (right) of prostate cancer between the CDC20-low and CDC20-high groups.
6. Comparison of the survival probability of prostate cancer between the CDC20 expression and the molecular signatures.


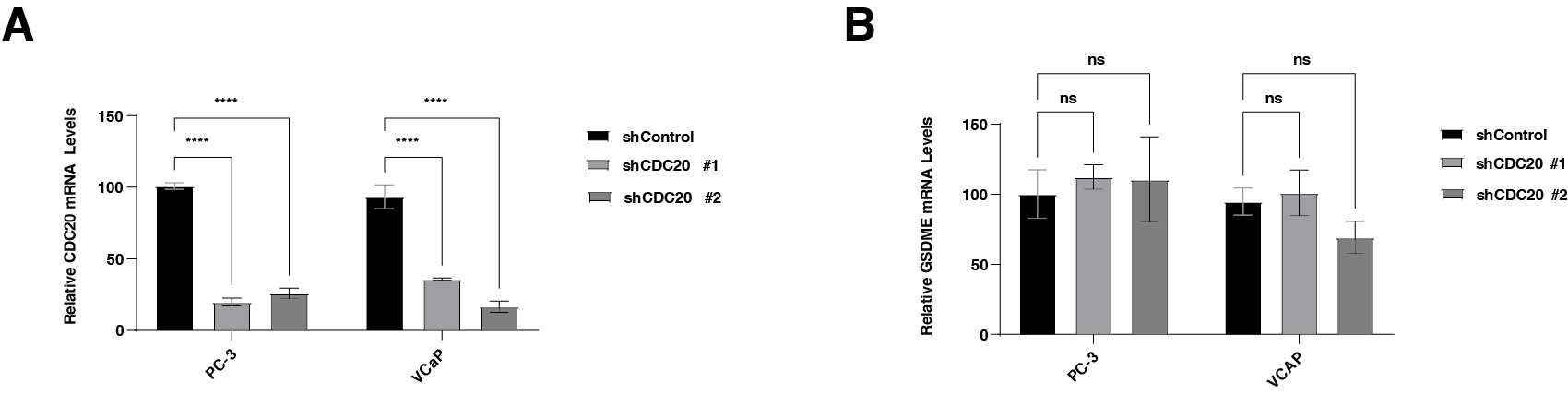


**Additional file 2: Fig. S2 Relative mRNA expression of *CDC20* (A) and *GSDME* (B) upon depletion of *CDC20***

**
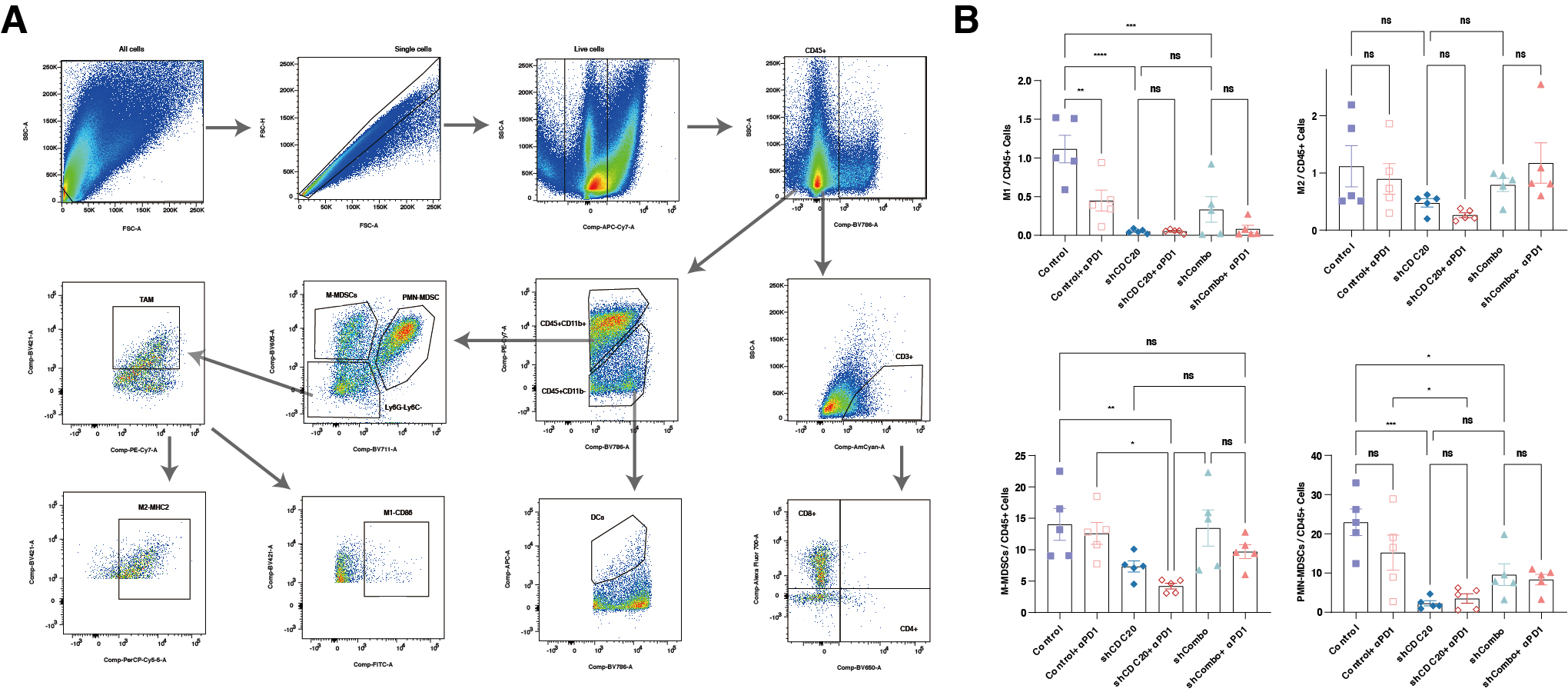
**

**Additional file2: Fig. S3 Fluorescence-activated cell sorting (FACS) gating strategy and analysis of the single cell suspension derived from TRAMP-C2 tumors**

1. Fluorescence-activated cell sorting (FACS) gating strategy.
2. Mice bearing TRAMP-C2 tumors infected with the indicated lentiviral shRNA constructs were implanted 7 days earlier and treated with or without anti-PD1 monoclonal antibodies (5 mice per group). Flow cytometric analysis of the single cell suspension derived from TRAMP-C2 tumors were performed. The proportion of M1-like macrophages to CD45^+^ cells, M2-like macrophages to CD45^+^ cells, PMN-MDSCs to CD45^+^ cells, M-MDSCs to CD45^+^ cells, and TAM to CD45^+^ cells were analyzed by FlowJo.


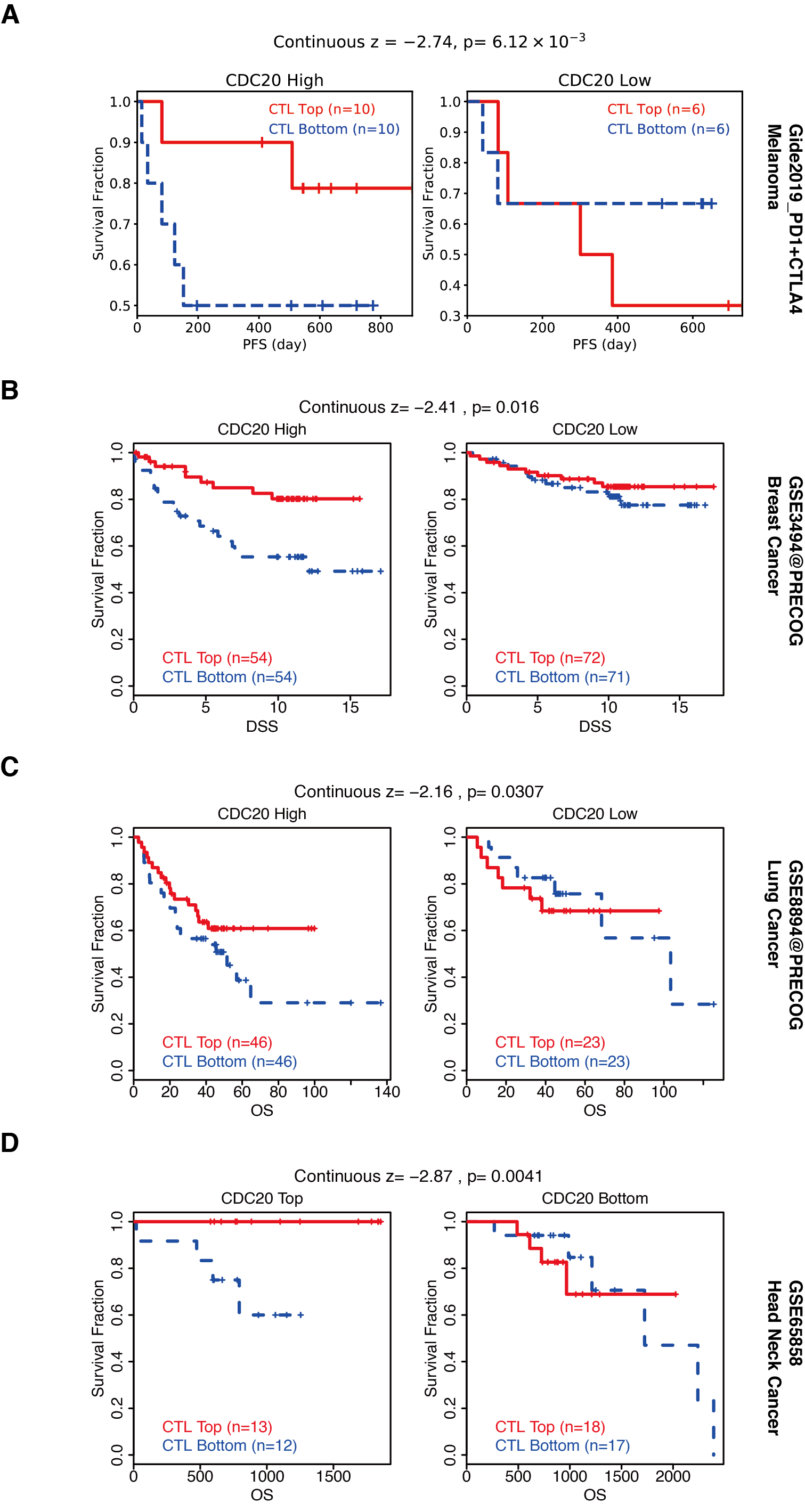


­­**Additional file2: Fig.S4 Relationship between survival fraction and CTL levels in CDC20-low and CDC20-high patients using TIDE.**

The figure displays the relationship between survival fraction and cytotoxic T lymphocyte (CTL) levels in two patient groups: CDC20-low and CDC20-high. Using TIDE (Tumor Immune Dysfunction and Exclusion), CDC20 expression profiles of each patient group to estimate their potential clinical benefits from immune checkpoint blockade therapy in patients with melanoma (A), breast cancer (B), lung cancer (C), and head and neck cancer (D).
